# In-line monitoring of latex-particle size during emulsion polymerizations with a high polymer content of more than 60%

**DOI:** 10.1039/d0ra02523b

**Published:** 2020-07-15

**Authors:** Laurence Isabelle Jacob, Werner Pauer

**Affiliations:** Institute for Technical and Macromolecular Chemistry, University of Hamburg Bundesstraße 45 20146 Hamburg Germany Werner.Pauer@chemie.uni-hamburg.de +49 40 42838 6007

## Abstract

The photon density wave (PDW) spectroscopy is established in the fields of biochemistry and food chemistry as an online analytical method for the determination of mean particle sizes. This work examines PDW spectroscopy regarding its potential in high solid content emulsion polymerization. For this reason, emulsion copolymerization with a tendency for agglomeration of vinyl acetate and Versa® 10 in a molar ratio of 9 : 1, and with varying emulsifier content, was carried out in semi-batch operation mode with different target particle sizes from 50 to 325 nm. A redox initiator system, consisting of l-ascorbic acid, *tert*-butyl hydroperoxide and ammonium iron(iii) sulfate, was used as a radical source. The mean particle sizes of PDW spectroscopy were compared with those of conventional offline measurement methods, such as dynamic light scattering (DLS) and sedimentation analysis, by means of a disc centrifuge. The determined mean particle sizes show a very good reproducibility and agreement between DLS and sedimentation analysis up to a polymer content of 36%, after which measurements were rendered difficult due to agglomeration. Nevertheless, PDW spectroscopy was able to continue providing reproducible measurements until reaching a polymer content of 63%.

## Abstract 2

The photon density wave (PDW) spectroscopy is established in the fields of biochemistry and food chemistry as an online analytical method for the determination of mean particle sizes.

## Graphical abstract

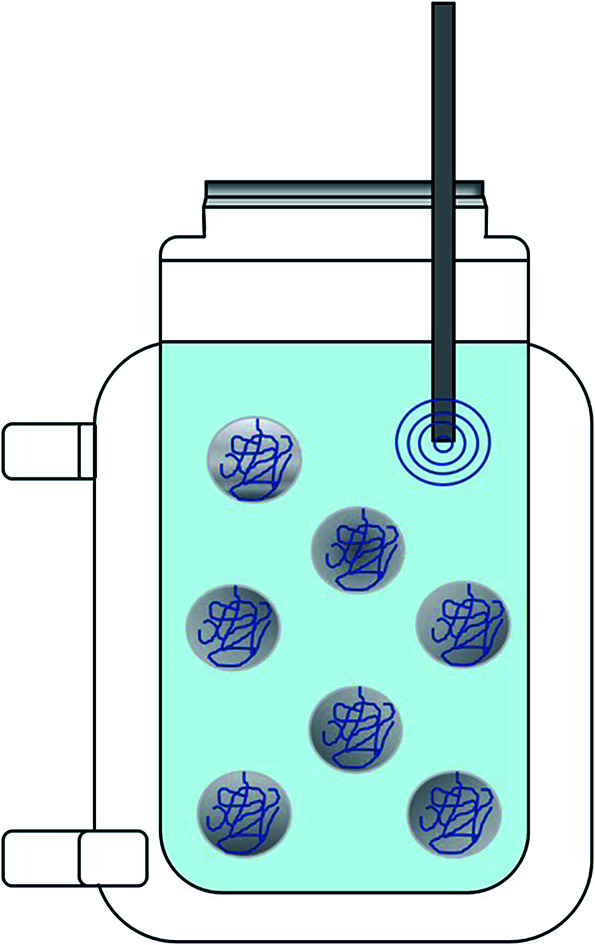

## Introduction

The importance of emulsion polymerisation in industry has continued to grow throughout the years, leading it to be the most commonly used process for the production of water-borne latex polymers.^1^ A commonly used monomer in the emulsion polymerisation is vinyl acetate, which is not only relevant in the industry, but also proved to be interesting for researchers.^2^ In order to ensure consistent product qualities, the produced dispersion has to be analysed thoroughly. The particle size distribution for example has, among other things, an influence on the surface properties of the dried polymer film or can provide information about the kinetics of the reaction as well as the number of radicals per particle.^3,4^ The development of the particle size must therefore be controlled in order to obtain the desired product.^5,6^ However, in order to achieve the necessary knowledge, it is essential to use the right analytical methods to determine reliably the particle size of the dispersion.

The most common methods for the determination of mean particle size and particle size distribution include dynamic light scattering^7^ and sedimentation analysis by means of a disc centrifuge. Both methods have in common, that the measurements are performed strictly offline. This of course has many disadvantages, as it requires a sample of the dispersion, which then has to be prepared. Most of the theories, on which these analytical methods rely, usually exclude high solid contents. For that reason, the sample must not only be prepared, but also be highly diluted. Each step involving a modification of the original dispersion may also alter the sample and therefore distort the results. The effort of sampling is not only time and money consuming, but it also carries the risk of inaccurate results, as well as altering the entire process, because of the disturbance. It is known, that polymers are products by process. By sampling during the reaction, the risk is increased, for example, of leaking oxygen into the vessel, which could inhibit the reaction. In order to avoid sampling, an inline measurement method would be preferable. Photon density wave (PDW) spectroscopy is currently being used in the fields of biochemistry and food analysis as an online analytical method.^8–13^ The method of PDW spectroscopy consists of measuring the reduced scattering coefficient of the dispersion. Because of the correlation between the scattering coefficient and the particle size, it is possible to then derive the latter. The device calculates the theoretical reduced scattering coefficient as a function of the particle diameter, which is why the PDW spectroscopy probe has multiple detector fibers at different but known distances from the emission fiber, thereby increasing the precision of the value. The measured reduced scattering coefficient is then used to determine the exact particle size of the dispersion, by measuring it at different wavelengths to assure the correct value. It is possible to use another number of wavelengths and detector fibers to perform the measurements, however, this would have an impact on the precision.^8–11,13–17^ It is a very efficient method, which is particularly well suited for dispersions with low absorption, and high scattering. Given that polymer dispersions fulfil these requirements, we examined PDW spectroscopy regarding its potential for high solid content emulsion polymerization.

## Materials and methods

All chemicals were used directly without further purification. Each component of the reaction was flushed beforehand with argon for at least 40 minutes.

### Emulsion polymerisations 20 wt%

The emulsion polymerisations were carried out in a 1.2 L double jacket glass reactor as a semi-batch process at 1 bar. The reactor used was 20.5 cm deep, had a diameter of 12 cm and a dish like bottom with the sample outlet in the middle. The reaction solution was stirred with a 45° pitched six blade stainless steel turbine with a diameter of 6 cm and at a rate of 300 rpm. The stirrer was located centrally and 3.5 cm above the bottom of the reactor. The initial charge contained 300–500 mL demineralised water, 0.06 g L^−1^ ammonium iron(iii) sulfate (Merck KGaA) as a catalyst and 0–75 g L^−1^ Mowiol 4-88 (Sigma-Aldrich) as an emulsifier, depending on the desired particle size to be achieved. The temperature of the reaction solution was brought to 60 °C within 30 minutes, while flushing its content with argon before starting the reaction. Four dosing units were used; the first contained the monomer mixture, consisting of vinyl acetate and Versa®10 (Wacker Chemie AG) in a molar ratio of 9 : 1. The monomers were fed at a rate of 4.5 g min^−1^.

The second dosing unit contained a 10 wt% Mowiol 4-88 solution, which was freshly prepared before each reaction. In order to achieve different particle sizes and particle size distributions, the rate of the second dosing unit was altered from 0.2–2 g min^−1^.

The third and fourth dosing units contained a 1.7 wt% solution of respectively the reducing and the oxidizing agents, being l-ascorbic acid (Sigma-Aldrich) and *tert*-butyl hydroperoxide (Sigma-Aldrich). They were fed at the same rate of 20 mL h^−1^.

The dosing occurred through PTFE hoses with an inner diameter of 3 mm. The end of each hose was immersed into the reaction solution, so that the dosing substance would be immediately stirred into the reactor content.

The catalyst was added to the reaction solution diluted in 1 mL demineralised water by using a polyethylene syringe from Henke-Sass, Wolf GmbH, just before starting the first dosing units. Then, the feeding of the reducing and oxidizing agents was started. After 5 min, the dosing of the monomer mixture and the emulsifier solution was started simultaneously and stopped after 1 h. The feeding of the reducing and oxidizing agents was continued for another 10 min after ending the dosing of the two other components before also being stopped.

The reducing and oxidizing agents were fed with a syringe pump from KD Scientific and using polyethylene syringes from Henke-Sass, Wolf GmbH. The monomer mixture and emulsifier solution were fed with Prominent Gamma/4 pumps with a maximum feed rate of respectively 0.2 L h^−1^ and 0.3 L h^−1^. The feed was regulated by a LabView program through Adlink NuDAM boxes and controlled by two Mettler Toledo GB3002 scales.

The temperature was regulated by a Julabo Cryo Compact F30-C thermostat.

When taking a sample, a 1.7 m% solution of hydroquinone (Sigma-Aldrich) was used to stop the reaction.

### Emulsion polymerisations 63 wt%

In order to achieve high solid contents of over 60%, the reaction needed to be carried out in four steps. All components were flushed with nitrogen for at least 40 min beforehand and the nitrogen flow inside the reactor was maintained during the whole process.

The emulsion polymerisation up to a polymer content of 20% was carried out the same way as described previously. After completing the reaction, the reactor was drained of half of its content, being replaced by the steady flow of nitrogen, then followed the second step of the reaction, being identical to the first, until reaching a polymer content of 30%. The reactor was drained again of half of its content, and the reaction pursued as in the first two steps until reaching a polymer content of 36%. After draining the reactor a final time of half of its content, the last step of the reaction was carried out, in which only the monomer mixture was fed to the reactor until reaching a polymer content of 63%.

**Fig. 1:**
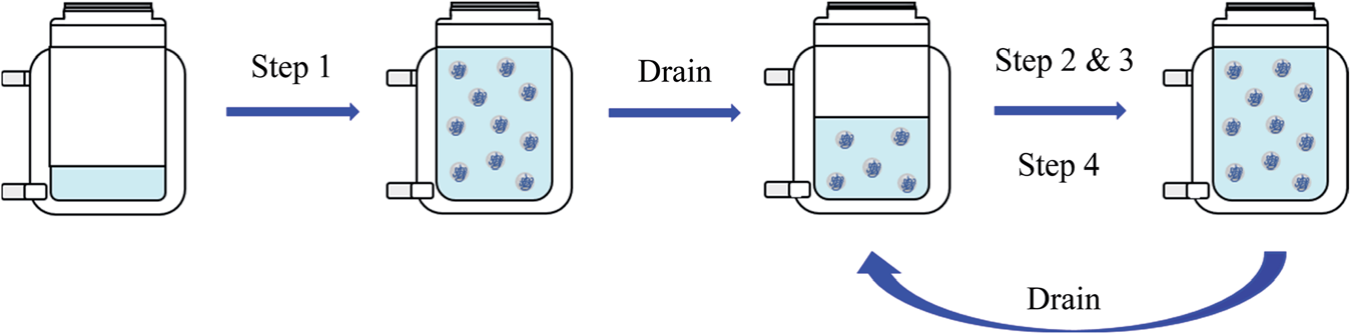
Summary of the four steps to obtain a dispersion with high polymer content. Step 1: Dosing of Mowiol 4-88 and monomer mixture up to a polymer content of 20%. Step 2: Dosing continued as in step 1 until reaching a polymer content of 30%. Step 3: Dosing continued as in step 1 and 2 until reaching a polymer content of 36%. Step 4: Dosing of monomer mixture only until reaching a polymer content of 63%.

Fig. 1 shows a simplified scheme of the four steps carried out during this reaction.

### Inline measurements

The PDW spectroscopy probe has a diameter of 2.5 cm and was placed centrally between the stirrer and the wall of the reactor, so that the probe protruded 1 cm into the initial reaction solution.

### Determination of yield

In order to determine the yield of each reaction, a sample was taken at the end of each reaction and measured by gas chromatography and by microwave analyser.

For the gas chromatography, 500 mg of the sample were mixed with 80 mg toluol as an internal standard and dissolved in 5 mL *N*,*N*-dimethylacetamide. The device used was an Agilent 7820A using hydrogen as a carrier gas.

For the microwave analyser, approximately 3 g of the sample were weighed, dried and weighed again, in order to determine the amount of solid contained by the dispersion. The device used was a Smart System 5 of the brand CEM.

### Determination of mean particle size

The mean particle size was determined by sedimentation analyses, by means of disc centrifuge, by dynamic light scattering and by PDW spectroscopy.

Measuring by sedimentation analysis requires a gradient, which is why 0.2 mL of methanol were injected into the disc centrifuge while in halt. Then the motor was started, and when reaching the maximum speed, 15 g of demineralised water were added steadily. One drop of the sample was diluted in 0.3 mL demineralised water and 0.1 mL methanol. 0.1 mL of the diluted sample was then injected into the disc centrifuge. The device used was a Disc Centrifuge DC24000 of the brand CPS. The accuracy lies at ±0.5%.

For the dynamic light scattering, one drop of the sample was diluted in 1 mL demineralised water and measured with a Zetasizer Nano ZS from Malvern. Each value was obtained through a triple determination, consisting of 18 measurements each at 25 °C. The accuracy of the device lies at ±2%.

The measurements with the PDW spectroscopy were carried out inline, which means the probe had to lie in the reactor during the process. The probe was passed vertically into the vessel and maintained at equal distance between the stirrer and the wall. The probe was far enough within the vessel for the sensor to immerse approximately 1 cm into the initial charge. The sample was exposed alternatively to light of four different wavelengths being at 638 nm, 781 nm, 915 nm and 961 nm. The device used was the Mini-PDW-spectrometer from the company PDW Analytics GmbH in collaboration with the InnoFSPEC of the University of Potsdam. The measurements were processed with software based on Labview 2016.

All measurements were made taking into consideration the previously examined refractive index as well as density of the copolymer. The density of the copolymer was determined with a DM45 from Mettler Toledo and the refractive index with a DSR-L from Schmidt & Haensch.

## Results and discussion

All experiments which were used for the comparison of the methods had a yield of at least 96% and showed a monodisperse particle size distribution.

### Correlation between the measurement methods

In order to attest the suitability of the PDW method, the measured particle diameters were plotted against each other. A linear fit of the results comparing PDW spectroscopy with a disc centrifuge gave a slope of (0.94 ± 0.06) and a *Y*-intercept value of (7 ± 11) nm. The fit has a correlation quality of 98% and a corrected *R*^2^ of 95%. This corresponds to a very good agreement of the data. The linear fit of the comparison between PDW spectroscopy and DLS gave a slope of (0.99 ± 0.14) and a *Y*-intercept value of (−62 ± 25) nm. The correlation quality of the fit is 92% and the corrected *R*^2^ is 85%.

**Fig. 2:**
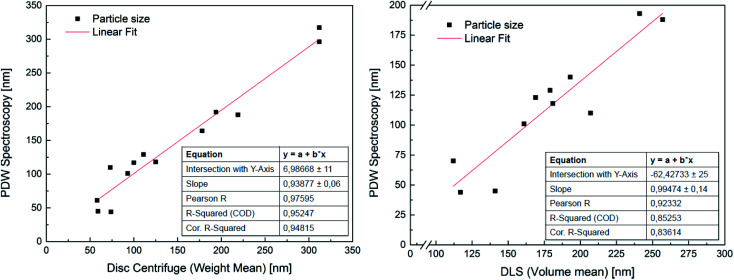
Correlation between PDW spectroscopy and sedimentation analysis by means of disc centrifuge (on the left) and dynamic light scattering (on the right).

**Fig. 3:**
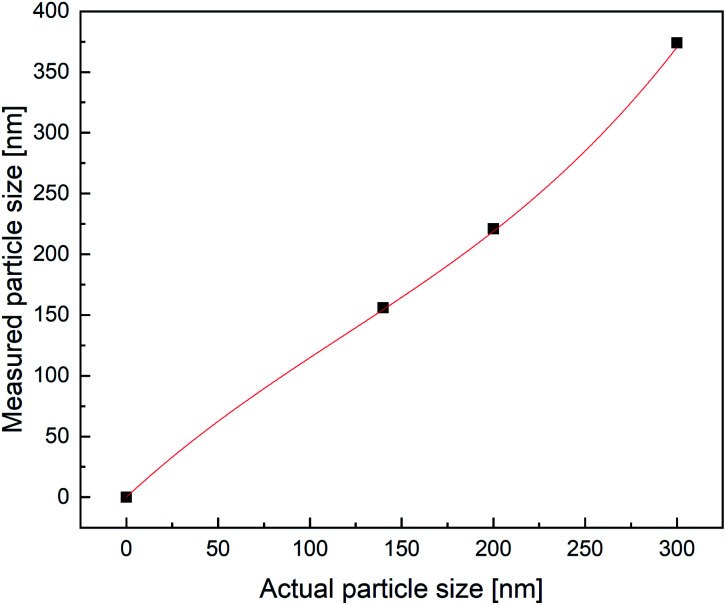
Calibration of the DLS device.

As shown in Fig. 2, there is a good correlation between the results obtained with all three methods. The average difference between the measurements with disc centrifuge and PDW spectroscopy lies at ±15 nm, although the majority show a difference of less than 10 nm. However, the average difference between the measurements with DLS and PDW spectroscopy lies at ±74 nm, but the overall offset on the *Y*-axis of −62 nm shows, that the average mean particle size measured by dynamic light scattering is approximately 62 nm bigger than by measuring with one of the other two methods. Previously published research showed, that the presence of polyvinylalcohol as protective colloid leads to “clathrate-like” structure promotion, which possibly makes the particle seem much larger, than it actually is, by measuring by dynamic light scattering.^18,19^ Although while the measurements by DLS are possibly affected by this, instead of measuring the hard shell, there also seems to be an offset of the device. This could later be proven by measuring three dispersions with a standard particle size distribution and comparing the measured particle size with the actual size of the particles in the standard dispersion. This experiment showed that the offset of the device grows larger with bigger particles (see Fig. 3). All measured particles by the DLS were then corrected following the fit of the new calibration, being:*y* = *A* + *B* × *x* + *C* × *x*^2^ + *D* × *x*^3^with *A* = −5.6275 × 10^−14^, *B* = 1.39684, *C* = −0.00346, *D* = 9.72321 × 10^−6^.

Considering this, the average difference between DLS and PDW spectroscopy measurements matches the one between the disc centrifuge and PDW spectroscopy.

### Suitability of PDW spectroscopy measurements for samples with a high optical depth

After establishing the comparability of PDW spectroscopy against DLS and sedimentation analysis, by means of a disc centrifuge, further experiments were carried out to examine the suitability of the method as an inline measurement for emulsion polymerisations at high solid contents.

**Fig. 4:**
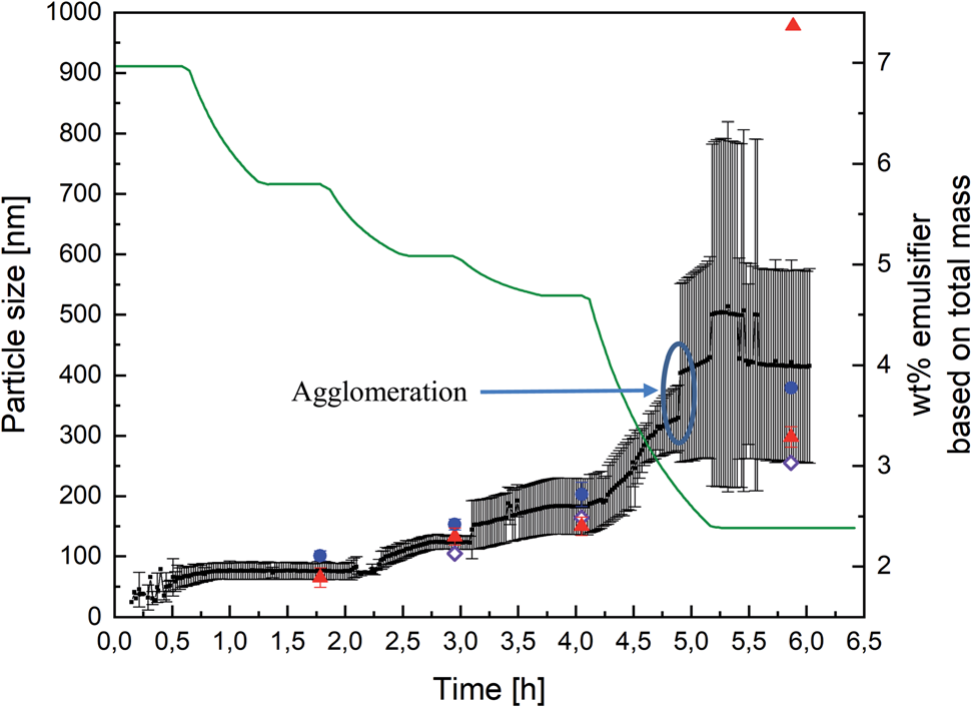
Record of PDW spectroscopy measurements (black dots) during all four steps of an emulsion polymerisation leading to a solid content of 63 wt%, with the standard deviation calculated by the software (black lines), as well as DLS measurements (blue dots), sedimentation analysis by means of disc centrifuge (red triangle) and the calculated particle size which should theoretically by obtained (purple diamond). The different steps of the polymerisation can be followed by looking at the total amount of emulsifier, based on the total reaction mass (green line). Each plateau equals a pause in the polymerisation in order to drain half of the reactor, as explained in “Materials and Methods” (see Fig. 1).

The experiments were carried out as explained before in “Materials and Methods” (see also Fig. 1). Measuring with PDW spectroscopy gives a value for the particle size approximately every minute of the reaction. The record of one exemplary reaction is shown in Fig. 4 with an overview of the measured particle sizes being shown in Table 1. After each step of the reaction, so after achieving respectively 20, 30, 36 and 63 wt% of polymer based on the total mass, diluted samples were measured by DLS and disc centrifuge to see, whether the results still correlate at high solid contents. Fig. 4 shows a very good agreement of the three measurement methods until achieving 36 wt%. At this point the dosing of the emulsifier was stopped, while the monomer dosing continued. As a result of achieving high solid contents, the particles started to agglomerate, which is why the further measurements are no longer in line with each other.

**Table 1:** Overview of the measured latex particle sizes from Fig. 4

Wt%	Calculated, nm	DLS, nm	Standard deviation, nm	Disc centrifuge, nm	Standard deviation, nm	PDW spectroscopy, nm	Standard deviation, nm
20	—	101	8	65	16	75	10
30	105	153	9	133	14	123	11
36	164	203	20	150	15	182	46
63	255	379	—	557/298	17	416	150

**Fig. 5:**
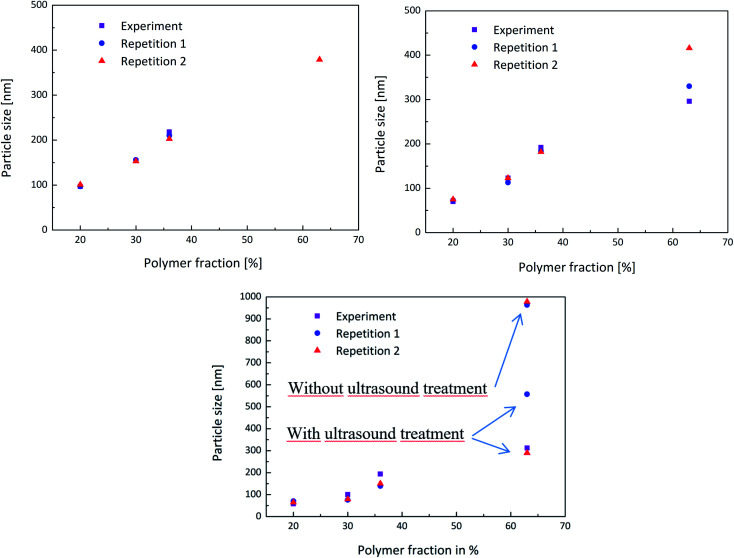
Reproducibility of the experiments shown by the repetition of the same procedure twice to compare the obtained measurements of DLS (top left), PDW spectroscopy (top right) and disc centrifuge (bottom).

In order to obtain a better overview whether the experiments were reproducible or not, the experiment above was carried out multiple times and the measurements then compared. Each PDW spectroscopy measurement showed a reliable record of the development of the particle size during the experiment and following the process. The reproducibility is shown in Fig. 5 and shows how each experiment provided the same results in each measurement method up to a polymer content of 36%. When achieving a high polymer content of 63%, the reproducibility of the experiment is no longer entirely accurate. This could be due to the formation of agglomerates, which highly disturbed the process, as well as the measurements. Nonetheless PDW spectroscopy was able to show at what point the agglomeration starts, so that the reaction can either be interrupted before agglomeration starts, or could be improved in the future.

Measuring with DLS or a disc centrifuge also proved to be a challenge after achieving such high polymer contents. Even though the samples were diluted, only once did the DLS manage to provide a realistic result. The disc centrifuge also seemed to have an issue with the agglomerates, which could however be improved by ultrasounding the sample beforehand. Fig. 5(c) shows that measuring the sample without ultrasound treatment leads to a very high particle size of approximately 1 μm, while measuring the same sample again, after a short ultrasound treatment improved the value to a more realistic particle size.

All latex particle sizes have been summarized in the following Table 2 for the DLS, in Table 3 for the PDW spectroscopy and in Table 4 for the disc centrifuge measurements.

**Table 2:** Overview of the reproducibility of the experiment regarding the latex particle size measurements of the DLS

DLS, wt%	Experiment, nm	Repetition 1, nm	Repetition 2, nm
20	97	97	101
30	154	156	153
36	218	210	203
63	—	—	379

**Table 3:** Overview of the reproducibility of the experiment regarding the latex particle size measurements of the PDW spectroscopy

PDW spectroscopy, wt%	Experiment, nm	Repetition 1, nm	Repetition 2, nm
20	70	73	75
30	123	113	123
36	192	184	182
63	296	330	416

**Table 4:** Overview of the reproducibility of the experiment regarding the latex particle size measurements of the disc centrifuge

Disc centrifuge, wt%	Experiment, nm	Repetition 1, nm	Repetition 2, nm
20	58	70	65
30	100	76	79
36	194	139	150
63	312	963	978
After ultrasound	—	557	298

### Influence of fouling

**Fig. 6:**
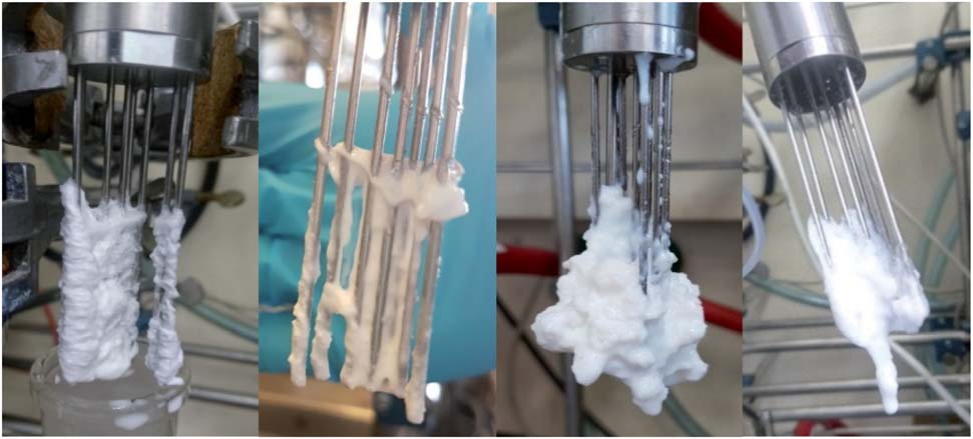
Fouling on a PDW spectroscopy probe. Both photos on the left show fouling which does not affect the measurement. The photos on the right show fouling which does have an influence.

PDW analytics asserted, that measurements with PDW spectroscopy are not influenced by fouling, in comparison to other methods such as turbidity measurements or Raman spectroscopy. While the current shape of the PDW spectroscopy probe is highly prone to formation of fouling (see Fig. 6), it had very little influence on the measurements. Less than 5% of the measurements had to be briefly interrupted in order to remove excess fouling which was placed directly on the optical fibres and therefore disturbed the measurements. After a quick removal the measurement went straight back to being as predicted.

## Conclusions

The suitability of PDW-spectroscopy was proven experimentally with a polymer content of up to 63 wt%. The measurements were reproducible and correlated very well with standard measurement methods such as dynamic light scattering and sedimentation analysis by means of disc centrifuge.

Additionally we were able to establish, that fouling has very little influence on the measurements.

However, PDW spectroscopy seems limited by the fact that the device requires a minimum scattering from the dispersion in order to be able to provide satisfying results. Moreover the method relies on the assumption of an infinite material availability, which would mean that no photons are lost due to collision with other materials. To be able to provide correct measurements, a certain amount of product is therefore required.

## Conflicts of interest

The authors declare no competing financial interest.

## Supplementary Material
